# Impact of embedded librarianship on undergraduate nursing students’ information skills

**DOI:** 10.5195/jmla.2021.913

**Published:** 2021-04-01

**Authors:** Susan R. Franzen, Jennifer Sharkey

**Affiliations:** 1 srfranz@ilstu.edu, Interim Associate Dean of Public Services and Organizational Development, Illinois State University, Normal, IL; 2 jsharke@ilstu.edu, Illinois State University, Normal, IL

**Keywords:** embedded librarian, assessment, collaboration, nursing, undergraduate students

## Abstract

**Background:**

One-shot library sessions have numerous drawbacks; most notably, they rarely have a long-term impact on students’ research behavior or skill sets. Library literature notes that when students interact with an embedded librarian, their skills improve. While close partnerships with subject faculty are important, librarians must also assess students’ skill sets to determine the impact of these teaching efforts.

**Case presentation:**

During the course, the embedded librarian used various activities and assignments to teach information-seeking skills, with the expected outcome of increased skill sets. This IRB-approved research project focused on measuring and assessing students’ information-seeking abilities before and after interacting with the embedded nursing librarian. Changes in students’ information fluency skills were measured using pre- and post-tests.

**Conclusions:**

The study results provide evidence of the benefits of the embedded librarianship model. Continued measurement of students’ skills acquisition is important to enable librarians and library administrators to show the positive impacts the library has on student learning and success.

## BACKGROUND

In “Roles and Strengths of Teaching Librarians,” the Association of College and Research Libraries (ACRL) defined seven major roles for a teaching librarian [1]. Two of these roles are “Teacher” and “Teaching Partner.” The role of “Teacher” encompasses many aspects of instruction, most of which are very difficult to incorporate in a “one-shot” session. Studies show that students do not retain information fluency skills when they are delivered in a “one-shot” format [2–6]. Instead, a teaching librarian should analyze the needs of each group, create a positive, interactive classroom, and engage in assessment. The role of “Teaching Partner” emphasizes the importance of collaboration with campus colleagues, including “build[ing] mutual trust” and “develop[ing] a shared vision.” Unfortunately, our academic colleagues do not often know the full extent of what librarians can offer, especially those traits described in the ACRL “Teaching Librarians” document. Embedded librarianship offers opportunities to share these skills with subject faculty. Embedded partnerships are librarians’ opportunity to demonstrate their value to their academic colleagues by investing more fully in the university curriculum and specific academic coursework.

In 2004, Barbara Dewey described embedding as “direct and purposeful interaction.” That purposefulness “makes embedding an appropriate definition of the most comprehensive collaborations for librarians in the higher education community” [7]. David Shumaker goes on to add that embedded librarianship is “a distinctive innovation that moves the librarians out of libraries and creates a new model of library and information work” [8]. Embedded librarians can be especially effective when fully immersed in the curriculum. They become part of a team of campus experts and offer unique, impactful contributions above and beyond a librarian's typical work [8]. Partnerships with subject faculty are crucial to integrate information fluency concepts within a course.

For embedded librarians to be successful, they must have strong partnerships with course faculty members who are content experts [9–11]. The most successful partnerships result from respect and trust. The faculty expert must see the librarian as an equal who is just as invested in the success of the students. When the partnership between the course instructor and librarian is built on mutual respect, the librarian can be viewed as a co-instructor rather than just an additional resource. The good opinion of the course instructor also translates into increased appreciation among students. Blake et al. found that, after the embedded experience, 84% of students would contact the librarian with questions about future projects [12]. Students better recognize the legitimacy of the librarian and understand their full scope of expertise when the instructor publicly supports the authority of the embedded librarian.

As Edwards et al. describe, librarians can be embedded in a variety of settings, including within colleges and departments, in face-to-face or online classrooms, and in research and writing projects with subject faculty [13]. Wu and Thornton describe a fully embedded librarian who works within a user group rather than in the library building and is considered part of the liaison department team [14]. In the health sciences, librarians are embedded in all these ways, although the most common is online embedding in course management systems [14–18].

Assessment is crucial to the success of library instruction. Embedded librarianship offers more opportunities to assess the work of students and the impact of the librarian. In Erlinger's review, collaboration is an important aspect of undergraduate information literacy instruction assessment [19]. Collaborative relationships with faculty lead to opportunities to evaluate the information-seeking skills of students. The students see the legitimacy of the librarian as an academic partner if the librarian is involved in assessment.

Embedded librarians can also assess the effectiveness of their instruction, and they can use student outcomes to adapt or change classroom lessons and/or activities. Edwards et al. use pre- and post-assessment to measure students’ self-efficacy as well as their information literacy skills [13]. In addition, they encourage embedded librarians to measure their contributions because “assessment is essential for evaluating program success” [13]. We followed this charge by designing a study to measure the impact of an embedded librarian in a face-to-face nursing research course with a course management system component.

## CASE PRESENTATION

### The course

Undergraduate nursing students at a Midwestern public university take a required class as juniors that is focused on evidence-based practice (EBP) research and theory. The purpose of the course is to develop students’ abilities to provide evidence-based care to patients and improve outcomes in a rapidly changing healthcare environment. In this course, students are introduced to research methods, EBP, the Johns Hopkins Nursing Evidence-Based Practice: Model and Guidelines [20], research article analysis, and database searching. These students simultaneously take clinical courses in adult and maternal infant nursing as well as advanced-level pathophysiology and pharmacotherapeutics. Students use experiences from their clinical placements in developing a question using the Patient Intervention Comparison Outcome (PICO) framework and applying EBP to real life.

The course is mostly lecture-based, with interactive classroom assignments requiring students to apply knowledge to scenarios or academic research articles. The major outcome of the course is a group poster presentation. Due to the close partnership developed with the subject faculty member, the nursing librarian had autonomy in the classroom, responsibility to redesign a major course assignment, and a commitment to adding library instruction questions to the midterm and final exams.

### The students

Prelicensure nursing students are required to take this course. For the most part, they have completed their general education classes and, at the time of this research course, are enrolled in their first year of the nursing core curriculum. While the majority of students are earning their first undergraduate degree, a cohort of approximately 30 students who earned their bachelor's degree in another field also take the course as part of an accelerated nursing program.

### Librarian instruction and student interactions

Prior to conducting this study, the nursing librarian was already embedded in the nursing course for several semesters, during which she worked closely with the instructor to add and revise the library content and activities. The librarian incorporated a variety of approaches designed to increase students’ information-seeking skills for their semester-long EBP group project.

Since the librarian was not a nurse, she had not previously taken a nursing research course or conducted nursing research. To better understand the course content, the embedded librarian attended weekly classes for two semesters. Additionally, the librarian was added as a co-instructor in the course management system, which allowed her to see the course from the instructor's perspective. Becoming familiar with the content made her better able to answer students’ questions throughout the semester as well as make revisions to a major course assignment.

Library instruction was meant to prepare students to be successful in completing a searching assignment called Search Strategy and Results (Appendix A), which required the students to delve into search strategy analysis. The Search Strategy and Results assignment is a major component of the semester-long project that culminates in a poster presentation. The librarian delivered two three-hour sessions of library content directly related to the searching assignment, which was due at midterm.

In the first library session, the focus was on search strategies, specifically for five different databases: Cumulative Index of Nursing and Allied Health Literature (CINAHL) Plus, PubMed, Joanna Briggs Institute EBP Database, Cochrane Library, and Turning Research into Practice (TRIP). During this session, students completed the librarian-created Search Strategy Organizer (Appendix B) to search for evidence to answer their PICO question. During this activity, the students used terms from their PICO question to locate articles using both keyword and subject heading search strategies in either CINAHL Plus or PubMed. The student group members then reviewed the search results and selected at least two articles they believed would help them address their PICO question. This classroom activity served as a foundation for the graded Search Strategy and Results assignment.

In the second three-hour session, the students focused on using and understanding the information found during their searching sessions. In class, the librarian reviewed the evidence rating scales from the Johns Hopkins Nursing Evidence-Based Practice: Model and Guidelines. Students worked in groups to evaluate one of the articles for their final poster project, using guided questions about each part of a scholarly research article. The librarian circulated to answer questions. The second half of the session focused on APA citation style, including a reference page containing errors and hands-on time using RefWorks. Outside of class, students utilized an online RefWorks help guide.

Due to the complexity of EBP research and to reinforce skills and concepts covered in both instruction sessions, the librarian also met with students in their small groups to provide targeted instruction for their specific PICO questions. The Search Strategy Organizer in-class activity performed in the first session prepared students for the group consultation. The students brought their search activity to the meeting with the two articles they selected. During the meeting, the librarian reviewed the two articles with the students, asking them questions about how they found them and what levels of evidence they were on the Johns Hopkins Nursing Evidence-Based Practice: Model and Guidelines. Students also asked questions about their search strategy and article types.

Based on her familiarity and involvement with the students’ search strategies, the nursing librarian requested to grade the Search Strategy and Results course assignment. The librarian did not use a rubric to assess the assignment, but rather used her expertise to evaluate the students’ skills to perform both keyword and subject heading searches as well as their ability to reflect on their own search strategies. This allowed her to see how the students were able to apply their knowledge. The librarian has plans to develop and use a rubric to ensure grading consistency and reduce time commitment. In addition, the librarian developed library content questions for the midterm and final exams.

### Information fluency skills instrument

To measure the impact of the embedded librarian on students’ information-seeking skills, the nursing librarian collaborated with the library's lead instruction librarian to develop an assessment instrument to be administered as a pre- and post-test. As a common assessment method, it has the advantage of more effectively showing an increase or decrease in knowledge [21]. Questions were adapted from a pre-existing internal instrument for the nursing research class and included queries about students’ skills in using health-related databases, finding nursing evidence, and evaluating research articles. The skills questions were formatted in typical objective test format using multiple choice. This format was chosen because it is a good method for determining gaps in knowledge [22]. The questions were aligned with the librarian's overall learning outcome of increasing students’ information-seeking skills.

Each possible answer was assigned a numeric value. The Skills Score was grouped as low (0–5), moderate (6–10), and excellent (11–15). Qualtrics was used to administer the pre- and post-test. Individual students were not tracked to maintain their anonymity per the IRB protocol. On the designated day, the librarian provided the link via the announcements page on the course site in the course management system. Student responses were completely anonymous and did not affect their assignment or course grades. As an alternative, a web-based assessment could be embedded into an HTML file or lesson module within a course management system. To maintain student anonymity, using Learning Management System quiz or testing tools are not recommended.

The pre-test was given at the beginning of the semester, usually during the second class period, before any planned library instruction or activities. Near the end of the semester, after library instruction, interactions with the librarian, and grading of their searching assignments, students completed the post-test. Over the course of three different semesters (two fall semesters and a spring semester), the nursing librarian asked students to complete the pre- and post-tests.

### Results of the pre- and post-tests

Since the number of participants varied by semester, we created a composite of all scores rather than break down the scores by semester. A total of 248 responses are represented in the composite data, with 155 responses to the pre-test and 93 responses to the post-test.

Students most frequently provided correct answers to Q5 in the pre-test (71%) and Q4 in the post-test (88%) (Table 1). The most difficult question in both the pre- and post-test was Q9, with only 11% and 13% of students providing correct answers, respectively. Students consistently chose the same wrong answer for this question, suggesting a problem with the question that requires further testing to remedy. Three questions included nursing databases as answers. In the pre-test, many students selected CINAHL for each answer regardless of whether it was the correct answer, indicating a familiarity with the database but lack of clarity about its scope. However, for these three questions, students chose the correct database more frequently in the post-test (Appendix C). Initial analysis of these data suggest that interactions with the embedded librarian had a positive impact on students’ skills. However, more data collection and analysis are needed to warrant a definitive conclusion.

**Table 1:** Correct responses for pre-test and post-test

Truncated Question	Pre-test	Post-test
Q1: Begin your search	22%	32%
Q2: Choose best database	45%	62%
Q3: Best source type for original data	27%	74%
Q4: Why use controlled vocabulary	68%	88%
Q5: What search terms are present	71%	80%
Q6: Most relevant source	37%	45%
Q7: Best search terms	15%	22%
Q8: Correct APA format	54%	76%
Q9: Best limiter in CINAHL	11%	13%
Q10: Best database to use	38%	57%

## DISCUSSION

As multiple instructors taught different sections of the course, the nursing librarian had to develop relationships with new subject faculty. In some cases, the new instructor of record wanted to continue with the same library activities and student interactions. In at least one case, the instructor asked the librarian to just lead a “one-shot” session even though the original instructor recommended the librarian and continued working closely with her in the other two sections. Subject faculty give many reasons for not wanting to develop close partnerships [23, 24], so the embedded librarian must take any opportunities given to deliver solid instruction in the hopes of creating a closer relationship with the faculty member in the future.

As has been discussed in the literature, the time commitment of embedded librarianship is a consideration [13, 16, 25]. While taking the class as a student helped the librarian understand the content, the time commitment was nearly overwhelming. If librarians are unable to devote time to taking the course, they can review course materials and reference the textbook as needed. The librarian found grading the searching assignment to be time-intensive, so after grading them for six semesters and helping to adapt the assignment based on student outcomes, she made the difficult decision not to grade the research assignment. Partnering on multiple sections with different faculty members was also a consideration in making that decision. In the future, a rubric could be used to ensure grading consistency and reduce the time commitment.

To gain the fullest possible picture of students’ skills and abilities, it is important to utilize a combination of assessments, including both practical (e.g., graded activities) and summative (e.g., written paper) assessments [19, 26]. The instrument used in this study was only one measure of students’ information fluency skills. While it proved invaluable in developing a snapshot of students’ abilities before and after librarian interactions, it did not show the full picture of progress or improvement. Additionally, pre- and post-tests do not reveal gaps in knowledge in real time. Since the embedded librarian had multiple contacts with students throughout a semester, she had opportunities to gather additional data points to show students’ skill level. One example was the grades of the searching assignment. While we chose to limit data gathering to just the pre- and post-tests, there is value in reevaluating ways to create a richer and deeper dataset.

Participation in the pre- and post-tests was optional. We found that the timing of its administration within a class session had an impact on number of responses. Students were typically given time at the end of class to complete the pre- and post-tests, and once finished they could leave. After a three-hour course, it was not uncommon for students to opt out of completing the questionnaire, as it meant getting out of class early. For purposes of future test administration, asking students to complete the pre- and post-tests at the beginning or in the middle of the class may increase participation.

In upcoming semesters, the nursing librarian will revamp her instruction to make it more interactive. In much the same way that virtually embedded librarians use discussion boards [13, 16, 25], she will give students opportunities to reflect on and discuss their search strategies in class. Using this feedback, the librarian will better know how to design additional activities to build on these student perceptions. Students continue to struggle with keyword and subject heading searching as well as understanding how to adapt a search strategy to find more relevant literature. By giving students more time in class to perform searches and explain their rationale for the strategies they use, the librarian can better prepare them to search the literature on their own time.

The librarian typically performed both three-hour library instruction sessions within the first five weeks of the semester. However, she would like to break up the three-hour sessions into shorter visits spread throughout the semester, especially closer to the time when the main poster project is due near the end of the semester. Data from the study conducted by Farrell et al. “indicated that students do indeed retain information literacy skills with an increased number of sessions” [27]. Thus, students will get library instruction closer to the time of need.

Consistency across sections would also prove beneficial. Since the research course is near the beginning of the students’ major nursing classes, they should all have the same experience with the librarian on whom they rely for future EBP projects. Standardizing the embedded librarian activities across all sections, regardless of the instructor, is a goal for the future.

## Data Availability

Data associated with this article are available in the Illinois State University Repository, ISU ReD, at https://ir.library.illinoisstate.edu/fpml/114/.

## SUPPLEMENTAL FILES

**Appendix A:** Search Strategy & Results Assignment

**Appendix B:** Search Strategy Organizer

**Appendix C:** Assessment Skills Questions
