# Supplementary material for: Impact of embedded librarianship on undergraduate nursing students’ information skills
Source: J Med Libr Assoc. 2021 Apr 1;109(2):311–6. doi: 10.5195/jmla.2021.913 (PMC8270371; doi:10.5195/jmla.2021.913)
Supplement: Supplementary file 1 — Appendix A: Search Strategy & Results Assignment [file jmla-109-2-311-s01.pdf]

Appendix A
Search Strategy and Results Assignment

This is a group assignment and will be done with your Evidence Based Practice (EBP) team. It is a **TWO-PART ASSIGNMENT**; submit the completed assignment in the Learning Management System. **Turn in papers with your EBP team name, names of all team members, and your PICO question on them. There are two parts to this assignment.**

PART 1: 3A (20 points)

General Directions: Complete your search for evidence, making refinements in your search terms as needed. You may use the directions on *Johns Hopkins Nursing Evidence-Based Practice (JHNEBP) Appendix B: Question development Tool* to guide you. Search at minimum the following databases:

- CINAHL
- Pubmed
- TRIP database
- Joanna Briggs OR Cochrane

You will do multiple searches in each database and note your results—your "hits"—as you complete in the table below.

1. Identify search terms based on your PICO question. Consider terms that are similar to those in your question.

   For example, if you are interested in adherence to medication regimen, you might consider also searching with the terms "compliance" and "concordance." Develop a search strategy – consider how you will actually conduct your search; what terms you will use and how you might combine them.

2. **Make an appointment to meet with the Nursing Librarian**. **Make sure you take your *Search Strategy Organizer*, in print, with you to the meeting!** Review your search strategy with her. Practice searching and refining your strategy.

3. First do a **keyword search** using words from your PICO question or phrases—this is the type of search you are used to doing on Google or other internet search engines. A keyword search is a broad search and may result in many articles that are irrelevant.

   *Remember, **apply limits** and filters to automatically restrict hits to those that meet minimum requirements—such as English language, published in the last 5 years, etc.*

4. Next do a **controlled vocabulary search** using *CINAHL headings* in CINAHL and *MeSH* in PubMed. Using subject headings, or the standardized language of the databases, will result in fewer but more focused results.

5. *Remember, **apply limits** and filters to automatically restrict hits to those that meet minimum requirements—such as English language, published in the last 5 years, etc.*

NOTE: Do not use "full text" as a filter for any of your searches

Complete the table noting the number of hits in each database for the keyword search, controlled vocabulary search, using limits/filters in each search. **(10 points)**

NOTE: Include PICO question on Assignment 3A

	**Keyword Search**<br>List keyword combinations, limiters used, & # of hits for each search.	**Controlled Vocabulary Search**<br>List subject heading combinations, limiters used, & # of hits for each
CINAHL	Search #1:<br>Search #2:<br>Search #3:	Search #1:<br>Search #2:<br>Search #3:
PubMed	Search #1:<br>Search #2:<br>Search #3:	Search #1:<br>Search #2:<br>Search #3:
Cochrane OR Joanna Briggs	Search #1:<br>Search #2:<br>Search #3:	
TRIP	Search #1:<br>Search #2:<br>Search #3:	
Other: (Optional)	Search #1:<br>Search #2:<br>Search #3:	

6. Skim the abstracts of your hits and select the best 10 to 15 pieces of evidence.
   a. If articles are available in full text, save to your computer.
   b. If you need to order articles through interlibrary loan, save articles to your computer when they arrive.
   c. If you need to copy articles from a print nursing journal, scan and save to your computer.

7. Write your search narrative in **essay form** (this should be at least a page and half in length). Make sure to answer each of the following questions in your essay:
   (10 points – 2 points per answer)

   a. **For each database**, what different combinations of terms did you use in your keyword search? Which were the most effective? Why?

   b. For CINAHL and PubMed, what controlled vocabulary words did you use for your searches and how did you find them?

   c. What limits/filters did you use **in each database** and how did you decide on these?

   d. How did changing your search strategy from keyword to subject heading as well as adding limits/filters impact the number of hits you got? Which of the searches (keyword or subject headings) was the most effective? Why?

e. How many articles/ pieces of evidence to appraise did you end up with at the end of your search? Why did you choose these articles instead of others (level of evidence, quality of evidence, focus on your topic, etc.)

NOTE: Strive to have at least 10, but no more than 16 articles/pieces of evidence to review.

Submit the searching table and narrative in the Assignment 3A dropbox in the Learning Management System.

PART 2: 3B (10 points)

Use RefWorks for organizing your group's articles. As a group, you will use ONE folder to collect and share sources with each other and your instructor. You will also use RefWorks to format a reference list in APA format.

1. Select one group member to create a folder in his or her personal RefWorks account. The **name** for the RefWorks folder must be your group's name.

2. Share the folder with your professor and the Nursing Librarian. In addition, share the folder with **all of your group members**.

   a. Watch the video tutorial to see how to create and share a RefWorks folder.

3. Create a subfolder in your group folder for PDFs of all articles. Add or drag and drop PDFs into the folder. **Make absolutely certain that a PDF of the actual article is available in Refworks**.

4. If the article is only available in HTML full text, you will need to convert it to pdf format. Most browsers have a Save as PDF option via the Print function. If you are not able to do this, you can use the free browser extension from PrintFriendly (https://www.printfriendly.com/extensions) to convert the file to pdf.

   a. Watch the video tutorial on how to drag and drop PDFs into RefWorks.

5. **Continue to use** this RefWorks folder to store all of the articles your group will read and use for the EBP project assignments.

6. **Develop a references list that includes the full reference** of all your selected articles IN APA FORMAT. **Points will be deducted** if APA format is incorrect.

7. Watch the video tutorial on how to create an APA reference list from RefWorks.

Submit this list in the Assignment 3B dropbox in the Learning Management System
