# Supplementary material for: Impact of embedded librarianship on undergraduate nursing students’ information skills
Source: J Med Libr Assoc. 2021 Apr 1;109(2):311–6. doi: 10.5195/jmla.2021.913 (PMC8270371; doi:10.5195/jmla.2021.913)
Supplement: Supplementary file 2 — Appendix B: Search Strategy Organizer [file jmla-109-2-311-s02.pdf]

Appendix B

Search Strategy Organizer

**What is your PICO question?**	
**Potential Search Terms from PICO Question:**	
**Database Used (CINAHL or PubMed):**	
**Keywords Used in Search:**<br><br>**Limiters:**	**Subject Headings Used in Search:**<br><br>**Limiters:**
**Article #1**<br><br>**Title**:<br>**Author(s)**:<br>**Journal**:<br>**Volume & Issue #s**:<br>**Pages**:<br>**Year**:	**Article #2**<br><br>**Title**:<br>**Author(s)**:<br>**Journal**:<br>**Volume & Issue #s**:<br>**Pages**:<br>**Year**:
**Summary/Key Points**:	**Summary/Key Points**:
