# Supplementary material for: Impact of embedded librarianship on undergraduate nursing students’ information skills
Source: J Med Libr Assoc. 2021 Apr 1;109(2):311–6. doi: 10.5195/jmla.2021.913 (PMC8270371; doi:10.5195/jmla.2021.913)
Supplement: Supplementary file 3 — Appendix C: Assessment Skills Questions [file jmla-109-2-311-s03.pdf]

Appendix C

Assessment Skills Questions

Q1: When you search for evidence for a class project, where do you BEGIN your search?

CINHAL Johanna Briggs Google Scholar
PubMed Librarian Browsing Library Stacks
Cochrane Milner/I-Share Catalog
Government Health web site (e.g. CDC, National Guideline Clearinghouse) Other

Q2: Which database would be the BEST one to find the source indicated?

	Systematic review	Practice Guidelines	Disease/Health Statistics	Open Source Research	Randomized Control Trials
CINAHL					
Joanna Briggs					
National Guideline Clearinghouse					
Google Scholar					
Center for Disease Control					

Q3: To support your PICO question, you need a source that presents and discusses original data gathered in a research study. Which type of source would be the best to use?

Case Study
Randomized Controlled Trial
Integrative Literature Review
Quality Improvement Project

Q4: Why is it a good strategy to use controlled vocabulary that represent the main ideas of your research question or statement?

It is a time saving technique so you only have to search once for information.
You will retrieve articles that are relevant to the main concepts of your topic.
Your search results will be displayed in a more organized manner.
You will retrieve only scholarly sources about your topic.

Q5: When looking for evidence-based journal articles on H1N1, you enter the keyword "Swine Flu" into a nursing database. What word(s) or phrase(s) will be present in every record of your search results?

H1N1
Swine Flu
H1N1 and Swine Flu
Influenza
Flu immunizations

Q6: You are doing an evidence-based research project on patient care practices for Alzheimer's patients. Which source listed below is the most relevant to your project?

Greb, E. (2012). Clinical Trial Results Highlight Diverse Ways to Treat Alzheimer's Disease. *Neurology Reviews*, *20*(10), 22–25.

Lava, N. (2017, April 23). Treatments for Alzheimer's Disease. Retrieved December 18, 2019, from WebMD website: https://www.webmd.com/alzheimers/guide/treatment-overview#1

Reuben, D. B., Roth, C. P., Frank, J. C., Hirsch, S. H., Katz, D., McCreath, H., … Wenger, N. S. (2010). Assessing Care of Vulnerable Elders—Alzheimer's Disease: A Pilot Study of a Practice Redesign Intervention to Improve the Quality of Dementia Care. *Journal of the American Geriatrics Society*, *58*(2), 324–329. https://doi.org/10.1111/j.1532-5415.2009.02678.x

Zarit, S. H., & Talley, R. C. (2013). *Caregiving for Alzheimer's disease and related disorders: Research, practice, policy*. New York: Springer. (Electronic Resources).

Q7: Statement: A recent Nurse Educator study reports that there is a correlation between more simulation lab time and fewer medication administration errors. You are interested in locating more research about this finding.

In relation to the statement above, what are the best search terms typed into a nursing database search box that would retrieve the most relevant results that represent the main ideas of the above statement?

Simulation, medication administration errors

Simulation, medication errors, nursing

More simulation lab time, fewer medication administration errors

More simulation lab time, fewer medication administration errors, correlation

Q8: Which citation is the correct APA format?

Diggle, J. (2015). The management of diabetes and best practice in injection technique. *Nurse Prescribing*, *13*(2), 72–78.

Diggle, Jane. "The Management of Diabetes and Best Practice in Injection Technique." *Nurse Prescribing*, vol. 13, no. 2, 2015, pp. 72–78.

Diggle, Jane. 2015. "The Management of Diabetes and Best Practice in Injection Technique." *Nurse Prescribing* 13 (2): 72–78.

Diggle J. The management of diabetes and best practice in injection technique. *Nurse Prescribing*. 2015;13(2):72-78.

Q9: What is the best method to use to limit a search in CINAHL to get high level, academic articles?

Use the Publication Type filter

Limit date range to the last five years

Limit to full-text

Use advanced search

Q10: If you have an interdisciplinary health-related question, which database would be best to use?

TRIP

Joanna Briggs database

PubMed

CINAHL
